# PRFdb: A database of computationally predicted eukaryotic programmed -1 ribosomal frameshift signals

**DOI:** 10.1186/1471-2164-9-339

**Published:** 2008-07-17

**Authors:** Ashton T Belew, Nicholas L Hepler, Jonathan L Jacobs, Jonathan D Dinman

**Affiliations:** 1 Department of Cell Biology and Molecular Genetics, University of Maryland, College Park, MD 20854, USA; 2 Laboratory of Molecular Growth Regulation, National Institute of Child Health and Human Development, National Institutes of Health, Building 6A/3A03, Bethesda, MD 20892, USA

## Abstract

**Background:**

The Programmed Ribosomal Frameshift Database (PRFdb) provides an interface to help researchers identify potential programmed -1 ribosomal frameshift (-1 PRF) signals in eukaryotic genes or sequences of interest.

**Results:**

To identify putative -1 PRF signals, sequences are first imported from whole genomes or datasets, e.g. the yeast genome project and mammalian gene collection. They are then filtered through multiple algorithms to identify potential -1 PRF signals as defined by a heptameric slippery site followed by an mRNA pseudoknot. The significance of each candidate -1 PRF signal is evaluated by comparing the predicted thermodynamic stability (ΔG°) of the native mRNA sequence against a distribution of ΔG° values of a pool of randomized sequences derived from the original. The data have been compiled in a user-friendly, easily searchable relational database.

**Conclusion:**

The PRFdB enables members of the research community to determine whether genes that they are investigating contain potential -1 PRF signals, and can be used as a metasource of information for cross referencing with other databases. It is available on the web at .

## Background

Canonical decoding of the genetic code requires translating ribosomes to convert triplets of bases (codons) into amino acid sequences. Although this is algorithm is employed for translation of the vast majority of mRNA sequences, in some special cases cis-acting mRNA elements direct ribosomes into alternative reading frames, dynamically "recoding" their sequence information (reviewed in [1]). Programmed -1 Ribosomal Frameshifting (-1 PRF) was first discovered in RNA viruses where it enables viral genomes to encode multiple peptides from a single mRNA [2]. An individual -1 PRF signal consists of a heptameric 'slippery site' usually followed by an mRNA pseudoknot secondary structure separated by a suitable spacer region (reviewed in [3-5]). Unlike their viral counterparts, eukaryotic genome-encoded -1 PRF signals are predicted to direct elongating ribosomes into premature termination codons (reviewed in [6]). Such events have been shown to initiate rapid mRNA degradation in yeast through the Nonsense Mediated Decay (NMD) pathway [7]. As such, -1 PRF is hypothesized to add a novel modality for regulation of gene expression at the post-transcriptional level.

There are currently three databases serving the translational recoding community. RECODE (please see Availability and requirements for more information) is a browsable collection of all the published translational recoding signals [8,9]. RECODE's strength is as central repository of all empirically proven translational recoding signals. FSDB (please see Availability and requirements for more information) contains a compilation of a handful of known and predicted viral, prokaryotic and eukaryotic -1 and +1 PRF signals, and also allows users to input their own sequences to search for frameshift signals using a program called FSFinder [10]. Although incomplete, this site provides tools not available through RECODE, in particular it integration of PseudoViewer, a powerful graphics tool that simplifies visualization of H-type pseudoknots [11]. MLOGD (please see Availability and requirements for more information) is a suite of software that allows detection of new protein-coding sequences by identifying overlapping open reading frames [12]. While all three of these sites have their strengths, a common weakness it that they do not provide well catalogued, searchable databases of all potential recoding signals of any one kind. To fill this gap, we have created PRFdb as a database of predicted -1 PRF signals in eukaryotic genomes. The methods used to search for predicted -1 PRF signals have been previously described, and importantly, we have empirically demonstrated that a significant number of -1 PRF signals so identified actually promote significant levels of frameshifting [13]. The strength of the PRFdb is that it provides a tool for researchers outside of the translational recoding field to use to quickly search for and identify potential -1 PRF signals in genes in which they are interested.

## Construction and content

In the PRFdb, the predicted -1 PRF signals are represented by: 1) the genes in which they reside; 2) the identity and location of their slippery sites; 3) graphical representations of their predicted secondary structures; 4) computationally identified minimum free energies (MFE); and 5) the thermodynamic significance of these mRNA structures as compared to randomized variants. Currently completed genomes in the PRFdb are *Saccharomyces cerevisiae *(88,683 sequences windows from 4,238 of 6,352 genes); *Homo sapiens *(24,104 sequence windows from 6826 of 17,891 genes); *Mus musculus *(19,313 windows from 6,053 of 15,620 genes); *Rattus norvegicus *(5924 sequence windows comprising 1982 of 5341 genes); *Bos Taurus *(10,690 windows from comprising 3349 of 9187 genes); *Danio rerio *(7640 sequence windows comprising 2618 of 6197 genes); and *Xenopus tropicalis *(9776 sequence windows comprising 2712 of 5126 genes). As of this writing, sequences from *Xenopus laevis *are being evaluated and entered into the database. The *Arabidopsis thaliana *genome is queued next in the pipeline.

**Figure 1:**
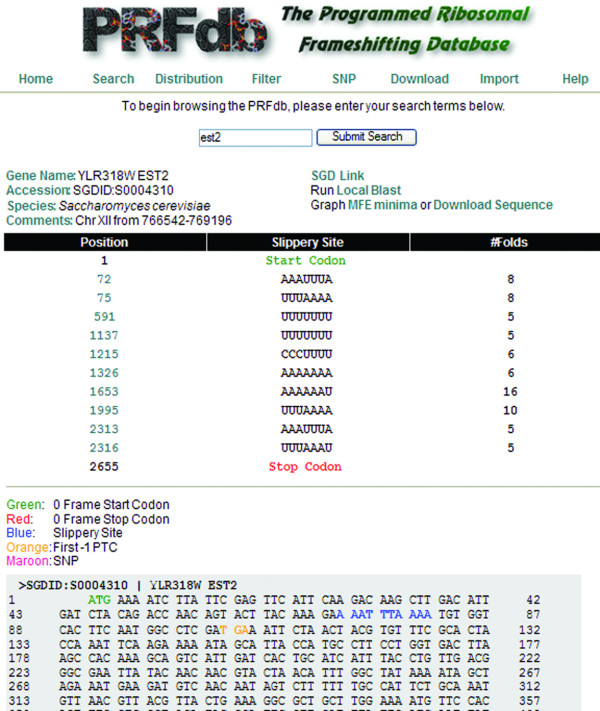
**Results when searching for the yeast *EST2 *gene**. The top of this page provides information pertaining to a specific gene (*S. cerevisiae EST2*), its genome database (SGD) entry, a link to perform *BLAST *searches for similar genes, MFE minima graph, and a link to download its sequence. Following this information is a list showing the locations of the translational start site, potential slippery sites and the number of secondary structure solutions that have been computed for them, and the 0-frame termination codon. At the bottom is a display of the gene where the ATG start site is displayed in green, slippery sites are shown in blue, and -1 frame termination codons are shown in orange. The specific entry for each potential frameshift signal may be viewed by clicking on the slippery site's position or its link in the sequence. In addition, locations of human single nucleotide polymorphisms catalogued in the NCBI Single Nucleotide Polymorphism Databaseare rendered in maroon, and clicking on these will open links to the database .

**Figure 2:**
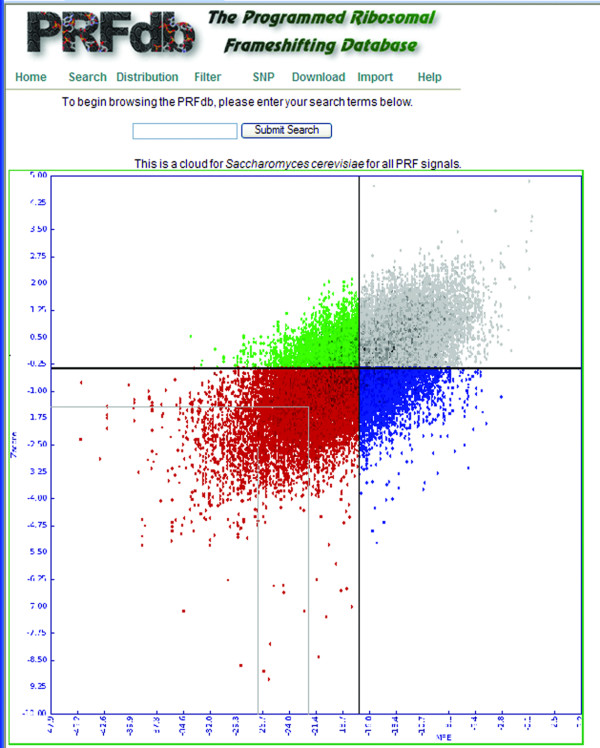
**The distribution of *Saccharomyces cerevisiae *sequences**. Computed minimum free energy is on the x-axis, ***z ***score is on the y-axis. Black lines denote the mean values and gray lines define sequence windows that are one and two standard deviations less than mean. Clicking on any region links to the closest -1 PRF signals with respect to MFE and ***z ***score.

Researchers can access data in the PRFdb through four means: *(i) Search *(Figure 1) provides a way to query a gene of interest using the specific gene name or description from the yeast genome project or mammalian gene collection. The search interface also provides a means to use BLAST to search for genes in the PRFdb similar to a query sequence. *ii) Distribution *(Figure 2) enables browsing for sequences containing statistically significant putative -1 PRF signals through a graphical representation of computed minimum free energies with respect to randomized ***z ***scores for all sequence windows. It is also possible to limit this distribution to sequences that are preceded by a specific slippery site. *iii) Filter *prints sequences from a given genome that meet specific criteria including: species, pseudoknotted sequence, sequences with a specific number of base pairs and/or MFE. *iv) Download *provides a format suitable for parsing all sequences of a given genome/sequence dataset.

**Figure 3:**
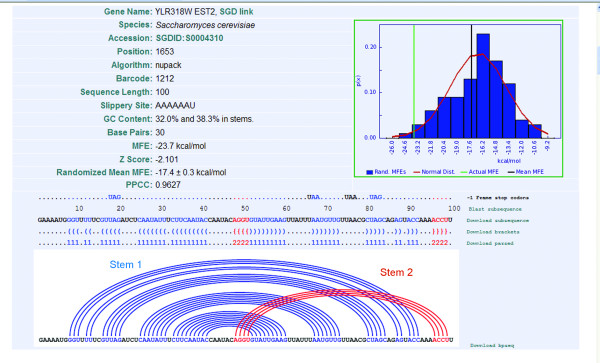
**The detailed interface**. This demonstrates that *pknots *was used to compute an MFE of -23.7 kcal/mol for the 100 bases following the AAAAAU slippery site at position 1653 of the *EST2 *gene. When randomized 100 times using Fisher-Yates shuffling, a mean MFE of -17.4 kcal/mol was computed for a normal distribution of correlation coefficient 0.9627. The MFE distribution of the randomized sequences is on the right; with the idealized normal distribution in red. The black vertical line marks the mean MFE of the randomized sequences, and the green vertical line marks the MFE of the native sequence. This secondary structure is significantly more stable than random (***z ***score = -2.10). The predicted mRNA secondary structure of this sequence is shown below using both bracket notation, and using a Feynman diagram.

The search, distribution, and filter interfaces lead to a detailed description of individual putative PRF signals (Figure 3). This provides a summary of all data gathered for a given sequence including: background information on the gene and location of the -1 PRF signal, information regarding the program used to perform the MFE prediction, multiple methods to view the secondary structure, and a comparison of the distribution of randomized sequences to the MFE of the folded sequence.

If a sequence of interest is not currently in the PRFdb, it can be imported via its NCBI accession number. Sequences added in this manner will be filtered within hours of import. Sequences imported into the PRFdb are also folded using sequential windows across the entire sequence in order to create a graphical minimum free energy 'landscape.' This enables users to submit longer or shorter sequence strings for computational folding, a particularly useful feature e.g. for eliminating extraneous sequence that may not be involved in actual RNA folding. For example, the computational analysis of the 100 nucleotide sequence downstream of the slippery site of the mouse Ma3 -1 PRF signal provided by the PRFdb predicts tandem stem loop structures. However, when only 55 nt of downstream sequence are provided, PRFdB predicts the empirically documented pseudoknot structure [14].

Sequences to be analyzed by the PRFdb are imported into the database, filtered using *RNAMotif *[15], folded with secondary structure prediction algorithms, randomized using one or more randomization methods, and refolded. To avoid complications of untranslated intronic sequences, the PRFdb contains only mature mRNA sequences (cDNA sequences primarily).

Sequences are imported into the PRFdb from a web interface using Genbank accession numbers, yeast genome accessions, or raw sequences. Each new sequence is first passed through a simple text filter that searches for slippery sites following the International Union of Pure and Applied Chemistry (IUPAC) pattern 'N NNW WWH' (or X XXY YYZ), where N (X) denotes any three identical bases, W (Y) denotes AAA or UUU, H (Z) ≠ G, and spaces indicate the incoming (zero) reading frame. Since the distance between the end of the slippery site and the downstream stimulatory sequence is important, a spacer of 1 to 8 nt was incorporated into the algorithm. Downstream sequences (100, 75 and 50 nt) are passed to *RNAMotif *with a descriptor looking for the potential to form an mRNA pseudoknot. Sequence windows passing this minimal test are passed to multiple pseudoknot predicting mRNA secondary structure prediction algorithms, including *Pknots *[16], *Nupack *[17], and *HotKnots *[18]. *Mfold *[19] and the Iterative Loop Matching algorithm are not used because *Mfold *does not predict pseudoknots and *ILM *does not provide the minimum free energy values for predicted secondary structures. After folding, every sequence is randomized using one or more algorithms including: Fisher-Yates shuffling to maintain dinucleotide frequencies, codon frequencies, or nucleotide frequencies. The resulting random sequence windows are then refolded without searching for pseudoknots. This process is repeated a fixed number of times (100 by default) to create a distribution of sequence specific randomized MFEs. These resulting distribution of randomized MFEs is then compared the MFE of the original sequence window. These values are used to compute a ***z ***score, thus providing a measurement of the significance of the native sequence.

## Utility

As with most bioinformatics projects, the generation of the PRFdB engenders more questions than answers. Most importantly, why is this database of useful to anyone outside of the frameshifting community? The response is that -1 PRF represents fundamental mechanism for the regulation of gene expression at post-transcriptional gene with far-reaching and broad impact. Thus, the PRFdB represents a resource that is useful and available to any researcher interested in how their gene or pathway of interest might be regulated at this level. Here, we have sought to simplify such inquiries by constructing a user-friendly web-based interface. The other major question concerns what the user might do following identification of a potential -1 PRF signal in a gene of interest. The classical "bench-based" approach that we are currently following is to clone the element into reporters and quantitatively determine whether it actually promotes -1 PRF (we typically use a cutoff level of ≥ 0.5% of a readthrough control), and whether it can function as an mRNA destabilizing element. Computational followup strategies include cross database referencing (e.g. assessing how -1 PRF in an mRNA or set of mRNAs might affect the transcriptome as monitored by microarray analysis), and phylogenetic analyses. In sum, the PRFdB provides yet another set of information for deep, one gene at a time mining, as well as for the broader approaches typified by the rapidly developing field of systematic biology.

## Discussion and conclusion

With regard to future development, the PRFdb is currently processing the *Xenopus laevis *genome. The *Arabidopsis thaliana *and *Caenorabditis elegans *genomes are currently in the queue, and additional genomes will be processed in the near future. In addition, the BLAST interface to the PRFdb has been used to discover Genbank sequences similar to the most statistically significant sequences in the database, thus providing a means to expand the PRFdb in a depth first manner. As more similar sequences are completed, comparative genomics studies using sequence and/or mRNA structure alignments will be incorporated to enable identification of conserved -1 PRF signals across species and/or genes. As time progresses, additional computational and empirical information will allow for improved scoring, helping to increase the statistical relevance of the predicted secondary mRNA structures. These improvements will continue to make the PRFdb more useful and accessible to the research community, providing a resource allowing individual users to identify -1 PRF signals in genes of interest, and as a metasource of information for cross referencing with other databases, e.g. genomes and DNA microarray databases.

## Availability and requirements

The PRFdb is freely available on the web at  and can be accessed though any standard web browser. Access and data downloading require no special requirements.

RECODE: 

FSDB: 

MLOGD: 

## Authors' contributions

The original process, code, and webpage design was undertaken by JLJ. ATB played the primary role in improving the original code, writing new code, improving the GUI and webpage design, and made significant contributions to the preparation of the manuscript. NLH wrote the code to incorporate SNP data into the PRFdb, and also helped to streamline operation of the database. JDD conceived and directed the project, and wrote and prepared the bulk of the manuscript. All authors read and approved the final manuscript.
